# Prescription of antibiotics and anxiolytics/hypnotics to asthmatic patients in general practice: a cross-sectional study based on French and Italian prescribing data

**DOI:** 10.1186/s12875-015-0222-0

**Published:** 2015-02-06

**Authors:** David Darmon, Laurent Laforest, Eric Van Ganse, Ferdinando Petrazzuoli, Chris van Weel, Laurent Letrilliart

**Affiliations:** Département d’enseignement et de recherche en médecine générale, Faculté de médecine, Université de Nice Sophia-Antipolis, 28 avenue Valombrose, 06 107, Nice, Cedex 02 France; Unité de Pharmaco épidémiologie, Faculté d’Odontologie, UMR 5558 CNRS – Université Claude-Bernard Lyon, CHU, Lyon, France; SNAMID Caserta (Italian Society of General Practice), Caserta, Italy; Department of Clinical Sciences in Malmö, Centre for Primary Health Care Research, Lund University, Malmö, Sweden; Department of Primary and Community Care, Radboud University Nijmegen Medical Centre, Nijmegen, The Netherlands; Australian Primary Health Care Research Institute, Australian National University, Canberra, ACT Australia; Département de médecine générale, Faculté de médecine, Université Claude-Bernard Lyon I, Lyon, France

**Keywords:** Asthma, Antibiotics, Anxiolytics, Hypnotics, Drug prescription, Primary care

## Abstract

**Background:**

Asthma is often poorly controlled and guidelines are often inadequately followed in medical practice. In particular, the prescription of non-asthma-specific drugs can affect the quality of care. The goal of this study was to measure the frequency of the prescription of antibiotics and anxiolytics/hypnotics to asthmatic patients and to look for associations between sex or age and the prescription of these drugs.

**Methods:**

A cross-sectional study was conducted using computerised medical records from French and Italian general practitioners’ networks. Patients were selected according to criteria adapted from the HEDIS (Healthcare Effectiveness Data and Information Set) criteria. The outcome measure was the number of antibiotics or anxiolytics/hypnotics prescriptions per patient in 1 year. Parallel multivariate models were developed.

**Results:**

The final sample included 3,093 French patients (mean age 27.6 years, 49.7% women) and 3,872 Italian patients (mean age 29.1 years, 48.7% women). In the univariate analysis, the French patients were prescribed fewer antibiotics than the Italian patients (37.1% vs. 42.2%, p < 0.00001) but more anxiolytics/hypnotics (17.8% vs. 6.9%, p < 0.0001). In the multivariate models, the female patients were more likely to receive antibiotics (odds ratio: 1.5 [1.3–1.7]) and anxiolytics/hypnotics (odds ratio: 1.8 [1.5–2.1]).

**Conclusions:**

The prescription of antibiotics and anxiolytics/hypnotics to asthmatic patients is frequent, especially in women. Asthma guidelines should address this issue by referring to other guidelines covering the prescription of non-asthma-specific drugs, and alternative non-pharmacological interventions should be considered.

## Background

Despite international guidelines [1], the proportion of patients with uncontrolled asthma remains high, independent of disease severity [2,3]. Among asthma-specific drugs, inhaled corticosteroids are often underused, and rescue medication is frequently overused [4,5]. Studies investigating insurance claims data have suggested that non-asthma-specific drugs such as antibiotics [6] or anxiolytics/hypnotics are widely prescribed to asthmatic patients [7] and to the general population [8,9]. The prescription of first-line antibiotics in primary care increases the population carriage of resistant organisms in the community and the use of second-line antibiotics [10]. Prescription of anxiolytics and hypnotics in primary care leads to addiction and other side effects such as daytime fatigue, ataxia, falls, and road traffic incidents [11]. Importantly, the actual prescribing of these drugs to asthmatic patients has been poorly studied in primary care practice. Direct comparisons of patients receiving and not receiving these prescriptions are lacking. This study aimed to measure the frequency of the prescription of antibiotics and anxiolytics/hypnotics to asthmatic patients and to look for any association between sex or age and the prescriptions of these drugs. We specifically compared the prescription profiles in general practice in two European countries with different healthcare systems for which comparable databases are available, namely France and Italy.

## Methods

### Data sources

We conducted a cross-sectional study using data from computerised French and Italian primary care databases. These two clinical databases, operated by Cegedim Strategic Data, collect consultation data from a network of 1200 general practitioners (GPs) (3% of French GPs) [12] distributed across France and a network of 700 GPs (1.3% of Italian GPs) [13] across Italy. Participating GPs continuously and voluntarily provide anonymised and coded patient data to a centralised database using an electronic health record system that is common within each country. In both countries, participating GPs are selected to be representative of the French and Italian populations, respectively, according to three main criteria: geographical area, age, and sex. Activity and prescription habits of the panels have also been compared with national data and shown to be representative [14-16]. This procedure has been approved in France by the National Data Protection Authority (ethics committee) since 2002 (reference number: 770334) and in Italy by the National Data Protection Authority since 2004 (no reference number). The data include patient demographic characteristics and the diagnoses and prescriptions related to each consultation. The quality of these databases has been checked regularly, and they have been used frequently for pharmacoepidemiological studies [17,18].

### Data extraction

The following variables were extracted from the databases: patient age and sex, number of visits to the GP and prescribed drugs, classified according to the Anatomical Therapeutic Chemical classification (ATC) [19]. Asthma drug categories belong to the ATC class R03 (Drugs for obstructive airway diseases) and include short-acting inhaled beta-agonists, long-acting inhaled beta-agonists (R03AC), oral beta-agonists (R03CC), theophylline (R03DA04), cromoglicic acid (R03BC), inhaled corticosteroids (R03BA), anticholinergics and leukotriene receptor antagonists (alone or in combination) (R03DC). We also extracted data on the use of oral corticosteroids (HA02AB). The following antibiotics were used for respiratory tract infections: tetracyclines (J01A), amphenicols (J01B), macrolides (J01F), beta-lactams (J01C), sulphonamides (J01E), cephalosporins (J01D), aminoglycosides (J01G) and quinolones (J01M). We also extracted data on all anxiolytics (N05B), hypnotics/sedatives (N05C), nasal preparations (R01) and antidepressants (N06A).

### Inclusion criteria

From a pre-selected group of patients with at least one R03 prescription in 2007, we included in the analyses all patients between the ages of 13 and 40 years on the first of January 2008 who consulted during the year 2007 or 2008 and who fulfilled the asthma criteria derived from Health Employer Data and Information Set criteria (HEDIS criteria) [20]. These criteria are based on prescriptions and are more accurate in retrieving data on asthmatic patients than criteria based on diagnostic label [21]. These criteria consisted of the prescription of four or more units of any ATC R03 class drug (alone or in combination), or four or more outpatient visits with a diagnosis of asthma and two or more drug prescriptions used in the treatment of asthma, within a year. Patients with any prescription of tiotropium bromide (R03BB04) in 2007 or 2008 were excluded, as well as those over 40 years old, to limit the risk of confusion with chronic obstructive pulmonary disease diagnosis.

### Statistical analyses

We used the issue of at least one prescription (one drug box versus none) as the outcome variable. First, the proportion of patients who received a prescription of at least one box of antibiotics or anxiolytics/hypnotics in 2008 was estimated for France and Italy. Then, these patients were compared with the other patients using a chi-square test for univariate analyses and a logistic regression model for multivariate analyses. These statistical analyses were performed using SAS 9.3 software (SAS Institute Inc., Cary, NC, USA). The selected level of significance was 0.05. In the univariate analyses, we estimated the prescription frequencies of at least one antibiotic and one hypnotic/sedative or anxiolytic in 2008. Using chi-square tests, we compared these frequencies between France and Italy and assessed the influence of patient age and sex, as well as the influence of the prescription of nasal preparations and antidepressants, as indicators of conditions frequently associated with asthma, such as rhinitis and depression.

Multivariate analyses were then conducted, based on logistic regression models after combining the French and Italian samples. We estimated odds ratios adjusted for asthma control criteria and severity (aOR), i.e. more than six prescriptions of short-acting inhaled beta-agonists (R03AC excluding R03AC12, R03AC13, R03AC14 and R03AC18) in 2008; the prescription of at least one inhaled corticosteroid (R03BA) in 2008, one inhaled asthma controller (R03AC12, R03AC13, R03AC14, R03AC18, R03AK06, R03AK07, R03BA, R03BC, R03DC, R03DA, R03DB or R03DX05), one oral corticosteroid (HA02AB) or the number of asthma drug units (R03; 1–7, 8–14, ≥15); and more than 12 visits to the GP with a prescription of an ATC R03 class medication in 2008.

## Results

**Figure 1:**
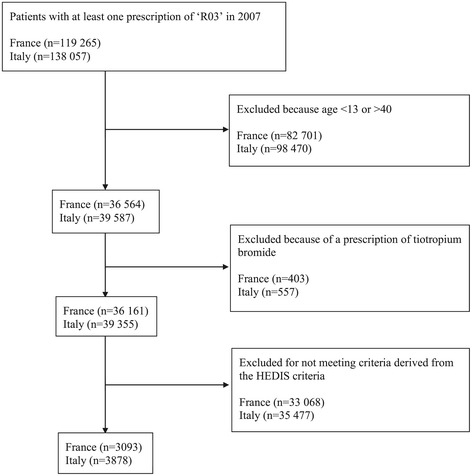
**Sample selection in France and Italy.**

The final sample included 3,093 French patients (mean age 27.6 years, 49.7% women) and 3,878 Italian patients (mean age 29.1 years, 48.7% women) (Figure 1). From our database, we estimated baseline proportions of 29.1% in France and 36.2% in Italy for non-asthmatic patients aged from 13 to 40 years in 2008 who were prescribed at least one box of antibiotics. For anxiolytics/hypnotics, the respective estimates were 9.5% in France and 3.2% in Italy. After adjustment for age, the prescription of antibiotics and anxiolytics/hypnotics was higher in asthmatic patients, as compared with non-asthmatic patients both in France (OR = 1.4, 95% CI [1.3–1.5] and OR = 2.1, 95% CI [1.9–2.3]) and in Italy (OR = 1.3, 95% CI [1.2–1.4] and OR = 2.2, 95% CI [1.9–2.5]) (Table 1).

**Table 1:** **Prescription of antibiotics and anxiolytics/hypnotics (at least one prescription in 2008) for asthmatic and non-asthmatic patients in France and in Italy according to patient age**

	**Asthmatic patient**		**Non-asthmatic patient**		
	**Yes**	**No**	**Yes**	**No**	**OR (95% CI)**
**Antibiotics**					
France					
Patients	1147 (37.1)	1946 (62.9)	190641 (29.1)	465037 (70.1)	1.43 (1.33-1.54)
[13-17]	169 (14.7)	316 (16.2)	24249 (12.7)	69162 (14.9)	
[18-29]	433 (37.7)	784 (40.3)	81854 (42.9)	211543 (45.5)	
[30-40]	545 (47.5)	846 (43.5)	84538 (44.3)	184332 (39.6)	
Italy					
Patients	1634 (42.1)	2244 (57.9)	96406 (36.2)	169550 (63.8)	1.28 (1.20-1.37)
[13–17]	180 (11.0)	238 (10.6)	10990 (11.4)	20334 (12.0)	
[18–29]	528 (32.3)	839 (37.4)	37915 (39.3)	65213 (38.5)	
[30–40]	926 (56.7)	1167 (52.0)	47501 (49.3)	84003 (49.5)	
**Anxiolytics/Hypnotics**					
France					
Patients	551 (17.8)	2542 (82.2)	62003 (9.5)	593675 (90.5)	2.08 (1.90-2.29)
[13–17]	28 (5.1)	457 (18.0)	3010 (4.9)	90401 (15.2)	
[18–29]	185 (33.6)	1032 (40.6)	24513 (39.5)	268884 (45.3)	
[30–40]	338 (61.3)	1053 (41.4)	34480 (55.6)	234390 (39.5)	
Italy					
Patients	267 (6.9)	3611 (93.1)	8454 (3.2)	257502 (96.8)	2.19 (1.93-2.48)
[13–17]	9 (3.4)	409 (11.3)	179 (2.1)	31145 (12.1)	
[18–29]	56 (21.0)	1311 (36.3)	2393 (28.3)	100735 (39.1)	
[30–40]	202 (75.6)	1891 (52.4)	5882 (69.6)	125622 (48.8)	

Data are presented as n (%) and OR denotes odds ratios adjusted on age and presented with their 95% confidence interval.

### Prescription of antibiotics

The proportion of asthmatic patients having at least one prescription of antibiotics in 2008 was higher in Italy than in France (42.1% vs. 37.1%, p < 0.0001). These prescriptions were more frequent in older patients in Italy and in female patients in both France and Italy (Table 2).

**Table 2:** **Prescription of antibiotics in France and in Italy (at least one prescription in 2008) according to patient age, sex, and nasal preparation and antidepressant prescriptions (univariate analyses)**

	**France**			**Italy**		
	**Yes**	**No**	**P-value**	**Yes**	**No**	**P-value**
**Patients**	1147 (37.1)	1946 (62.9)		1634 (42.1)	2244 (57.9)	
**Age (yrs.)**						
[13–17]	169 (14.7)	316 (16.2)	0.09	180 (11.0)	238 (10.6)	<0.01
[18–29]	433 (37.7)	784 (40.3)		528 (32.3)	839 (37.4)	
[30–40]	545 (47.5)	846 (43.5)		926 (56.7)	1167 (52.0)	
**Sex**						
Female	686 (59.8)	852 (43.8)	<0.0001	902 (55.2)	983 (43.9)	<0.0001
Male	461 (40.2)	1094 (56.2)		731 (44.8)	1256 (56.1)	
Nasal preparations prescription*						
No	293 (25.5)	1055 (54.2)	<0.001	1428 (87.4)	2007 (89.4)	<0.05
Yes	854 (74.5)	891 (45.8)		206 (12.6)	237 (10.6)	
Antidepressants prescription*						
No	1038 (90.5)	1831 (94.1)	<0.001	1503 (92.0)	2112 (94.1)	<0.05
Yes	109 (9.5)	115 (5.9)		131 (8.0)	132 (5.9)	

Data are presented as n (%). *At least one prescription in 2008.

### Prescription of anxiolytics/hypnotics

The proportion of asthmatic patients with at least one prescription for anxiolytics or hypnotics in 2008 was more frequent in France than in Italy (17.8% vs. 6.9%, p < 0.0001). The prescription of anxiolytics or hypnotics was more frequent in older and female patients in both countries (Table 3).

**Table 3:** **Prescription of anxiolytics/hypnotics (at least one prescription in 2008) in France and in Italy according to patient age, sex, and nasal preparation and antidepressant prescriptions (univariate analyses)**

	**France**			**Italy**		
	**Yes**	**No**	**P-value**	**Yes**	**No**	**P-value**
**Patients**	551 (17.8)	2542 (82.2)		267 (6.9)	3611 (93.1)	
**Age (yrs.)**						
[13–17]	28 (5.1)	457 (18.0)	<0.0001	9 (3.4)	409 (11.3)	<0.0001
[18–29]	185 (33.6)	1032 (40.6)		56 (21.0)	1311 (36.3)	
[30–40]	338 (61.3)	1053 (41.4)		202 (75.6)	1891 (52.4)	
**Sex**						
Female	359 (65.15)	1179 (46.4)		186 (69.7)	1699 (47.1)	
Male	192 (34.8)	1363 (53.6)	<0.0001	81 (30.3)	1906 (52.9)	<0.0001
Nasal preparations*						
No	222 (40.3)	1126 (44.3)	0.09	237 (88.8)	3198 (88.6)	0.92
Yes	329 (59.7)	1416 (55.7)		30 (11.2)	413 (11.4)	
Prescription of antidepressants*						
No	401 (72.8)	2468 (97.1)	<0.0001	169 (63.3)	3446 (95.4)	<0.0001
Yes	150 (27.2)	74 (2.9)		98 (36.7)	165 (4.6)	

Data are presented as n (%). *At least one prescription in 2008.

In the multivariate analyses (Table 4), the prescription of antibiotics was less frequent in France than in Italy (aOR = 0.8, 95% CI [0.7–0.9]) and more frequent in female than in male patients (aOR = 1.5, 95% CI [1.3–1.6]). The prescription of antibiotics was also associated with the prescription of nasal preparations (aOR = 2.0, 95% CI [1.7–2.2]). The prescription of anxiolytics or hypnotics was more frequent in France than in Italy (aOR = 5.0, 95% CI [3.4–5.3]), in female patients (aOR = 1.8, 95% CI [1.5–2.1]) and in older patients (aOR = 1.9, 95% CI [1.3–2.8] between 18 and 30 years and 3.3, 95% CI [2.3–4.7] between 31 and 40 years). The prescription of these drugs was associated with the prescription of antidepressants (aOR = 9.6, 95% CI [7.7–11.9]).

**Table 4:** **Prescription of antibiotics and of anxiolytics/hypnotics (at least one prescription in 2008) according to country, patient age, sex, and nasal preparation and antidepressant prescriptions, after adjusting for asthma control and severity criteria (multivariate logistic models)**

	**Model 1 Prescription of at least one antibiotic in 2008 (n = 6965)**	**Model 2 Prescription of at least one anxiolytic/hypnotic in 2008 (n = 6965)**
**Factors**		
France (vs. Italy)	0.76 (0.66- 0.87)	5.00 (3.45-5.26)
Age [18–29] (vs. [13–17])	0.98 (0.83-1.16)	1.92 (1.33-2.76)
Age [30–40] (vs. [13–17])	1.10 (0.93-1.30)	3.31 (2.33-4.72)
Female (vs. Male)	1.48 (1.34-1.65)	1.81 (1.53-2.14)
Nasal preparations*	1.99 (1.75-2.27)	0.98 (0.81-1.19)
Antidepressants*	1.22 (1.00-1.49)	9.56 (7.69-11.89)

Data are presented as adjusted odds ratios, with their 95% confidence interval.

*At least one prescription in 2008.

## Discussion

### Main findings

Using the data from two large computerised databases, we observed that non-asthma-specific drugs are commonly prescribed to asthmatic patients in France and Italy. In particular, in 2008, 37.1% of the French patients and 42.1% of the Italian patients were prescribed antibiotics, and 17.8% of the French patients and 6.9% of the Italian patients were prescribed anxiolytics or hypnotics. A higher frequency of prescription was observed for female patients for these two drug categories in both countries and in older patients for anxiolytics or hypnotics in France. These results are comparable to known rates of prescription for antibiotics, anxiolytics and hypnotics in non-asthmatic patients.

### Strengths and limitations

A strength of this study is the use of accurate drug prescription data from community-based general practice, where asthmatic patients are treated most of the time. Our data come from France and Italy, countries with poor documentation of primary care performance [22]. Our analyses are based on prescription data, not on claims data or on data on drugs dispensed over the counter. Although they do not capture the over-the-counter drug consumption or perfectly document adherence to prescribed drug regimens, these prescription data reflect the actual practice of French and Italian GPs [23]. Although we selected antibiotics typically indicated for respiratory tract infections, their prescription may have also been for reasons other than asthma exacerbation. We were unable to assess severity factors not readily available (such as socioeconomic factors or spirometry) or unreliable (such as smoking status) [24]. Because the derived HEDIS criteria are partly based on drug prescriptions to identify patients with persistent asthma, we may have underestimated the proportion of asthmatic patients in the GPs’ lists and the frequency of non-asthma-specific drug prescriptions [25]. We used as outcome criteria the prescription of at least one box of drug rather than the defined daily dose because the aim of the study was primarily to measure the frequency of prescription of antibiotics and anxiolytics/hypnotics.

### Antibiotic prescription

When presented with an asthma exacerbation, GPs are likely to overestimate the risk of bacterial infection and prescribe antibiotics [26,27]. This trend is associated with the underuse of inhaled corticosteroids, probably because of a misunderstanding of the immunosuppressive effect of corticosteroids [28]. However, antibiotics are frequently not useful in asthma exacerbations (for those caused by viral infections, for example), unless they are of bacterial origin or are associated with bacterial acute sinusitis in children [1,29]. A computed tomography scan is recommended for confirmation when sinusitis is suspected in adult patients [1]. However, because the clinical features of sinusitis lack diagnostic precision [30,31], GPs can face delays in getting access to a computed tomography scan for their patients and may prescribe antibiotics as a precautionary measure [32]. Independently, evidence is lacking on the actual value of the use of antibiotics, such as macrolide treatment for at least 4 weeks, in the treatment of chronic asthma [33]. The over-prescription of antibiotics to asthmatic patients may cause adverse events, increase costs, and contribute to the development of antibiotic resistance in microbes [17]. Interestingly, antibiotic use in the first year of life is a risk factor for asthma [34].

France and Italy belong to the group of European countries with a high level of antibiotic consumption (17–24 daily doses per day per 1000 inhabitants); France is currently ranked second and Italy third, behind Greece [35]. The small difference that we observed in the numbers of antibiotic prescriptions between France and Italy may be because such prescriptions are specific for respiratory tract infections. Our results confirm that women are prescribed more antibiotics than men [36,37], which may be owing to poorer control of their asthma, among possible reasons [3].

### Anxiolytic and hypnotic prescriptions

Our estimation of the frequency of prescription of anxiolytics or hypnotics in French asthmatic patients (17.8 %) is consistent with observations from the available dispensing data (25.6% for anxiolytics and 13.0% for hypnotics) [26]. In Italy, we observed less frequent prescription of drugs in this category (6.9%) compared with France. A possible explanation for this finding is that patients must pay for all benzodiazepine prescriptions [38]. Thus, a GP may preferentially prescribe other reimbursable drugs. The high prevalence of anxiety disorders and insomnia in France, especially in older people, may explain the high numbers of prescriptions for anxiolytics and hypnotics [39]. More specifically, these prescriptions may be related to poor asthma control [3,40-42]. This relationship could be interpreted in two ways: asthma exacerbation could induce anxiety [43] or psychiatric disorders could be risk factors for asthma exacerbations [44].

There are guidelines that promote the provision of psychological support to asthmatic patients and that recommend the limited prescription of psychotropic drugs [31]. The long-term use of anxiolytics and hypnotics should be avoided because of their addictive effect [45], and their use should be avoided during asthma exacerbations because of their respiratory depressant effects, which are potentially lethal [44]. Although cognitive-behavioural therapies focussed on worry proneness and the overvaluation of worry and uncertainty may be as effective as drug treatment and may be more durable for treating anxiety disorders [46], these results remain to be confirmed in asthmatic patients [47].

In Europe and North America, women are generally prescribed twice as many psychotropic drugs as men [48]. Psychiatric disorders and complaints are indeed more common in women than in men [49]. Women are also exposed to specific situations such as pregnancy, which can both worsen asthma and generate anxiety [26,50]. In addition, women have fewer opportunities than men to control symptoms of anxiety through social activities outside the home, including the use of alcohol [51-53].

### Implications for research, policy and practice

The high prevalence of antibiotic prescription in asthmatic patients suggests a likely antibiotic over-prescription in France and Italy. While anxiety is frequent in asthmatic patients, the prescription of anxiolytics and hypnotics should be avoided and the use of non-pharmacological interventions should be considered in these patients. Apart from international guidelines, there is only one French guideline from 2004 on long-term asthma management. The French guideline does not mention the prescription of antibiotics and anxiolytics/hypnotics, and there are no specific Italian recommendations on asthma management.

## Conclusions

While it is known that many asthmatic patients do not take specific controllers, the prescription of non-asthma-specific drugs is common, especially in women. Asthmatic patients often have additional health and life concerns. GPs should use safe interventions and take a biopsychosocial perspective. The prescription of antibiotics and anxiolytics/hypnotics to asthmatic patients should follow the same rules that apply to their prescription in non-asthmatic patients. Asthma guidelines should address this issue and refer to other guidelines covering the prescription of these drugs. Alternative non-pharmacological interventions should be considered.

## Abbreviations

aOR: Adjusted odds ratios
ATC: Anatomical Therapeutic Chemical classification
GP: General practitioner
HEDIS: Health Employer Data and Information Set
